# Response to “Rethinking Machine-Learning Metrics in Schistosomiasis Control: Toward Predictive Equity, Field Readiness, and Biological Foresight”

**DOI:** 10.4269/ajtmh.25-0470b

**Published:** 2025-09-30

**Authors:** Xinyue Chen, Jiaxu Le, Yi Hu

**Affiliations:** Department of Epidemiology and Biostatistics School of Public Health Fudan University Shanghai, China Key Laboratory of Public Health Safety Ministry of Education Shanghai, China Laboratory for Spatial Analysis and Modeling School of Public Health Fudan University Shanghai, China E-mail: 22211020119@m.fudan.edu.cn; Department of Epidemiology and Biostatistics School of Public Health Fudan University Shanghai, China Key Laboratory of Public Health Safety Ministry of Education Shanghai, China Laboratory for Spatial Analysis and Modeling School of Public Health Fudan University Shanghai, China E-mail: ljx9756@163.com; Department of Epidemiology and Biostatistics School of Public Health Fudan University Shanghai, China Key Laboratory of Public Health Safety Ministry of Education Shanghai, China Laboratory for Spatial Analysis and Modeling School of Public Health Fudan University Shanghai, China E-mail: huyi@fudan.edu.cn

We sincerely appreciate the thoughtful and constructive letter by Nathkapach Kaewpitoon Rattanapitoon, et al. regarding our recent publication, Predicting Schistosomiasis Intensity in Africa: A Machine Learning Approach to Evaluate the Progress of World Health Organization (WHO) Roadmap 2030.^1^ Their insights align closely with our team’s mission to develop practical and equitable solutions for schistosomiasis control, and we are grateful for the opportunity to address their valuable suggestions.

## Model interpretability and SHapley Additive exPlanations (SHAP) analysis.

We agree that high numerical accuracy alone is insufficient to ensure programmatic uptake and field utility. To enhance interpretability and operational relevance, we conducted a preliminary SHAP analysis using the 2020 dataset. The results indicated that predicted heavy-intensity schistosomiasis prevalence was primarily influenced by snail habitat suitability (41%), proximity to perennial water bodies (22%), and mean land surface temperature (18%). To support evidence-based decision-making in endemic settings, we plan to 1) develop and disseminate an interactive web-based dashboard that provides localized SHAP visualizations for each survey site, and 2) co-develop a concise policy brief in collaboration with focal points from the Expanded Special Project for Elimination of Neglected Tropical Diseases (ESPEN), aimed at translating model outputs into actionable intervention options (e.g., focal molluscicide and targeted health education). These resources will be made openly accessible via the ESPEN Collect platform to facilitate alignment with national control program planning and implementation.

## Biological complexity and snail host data.

The emphasis on ecological dynamics, particularly snail-host interactions and parasite diversity, is well taken. Although many site-specific surveys of snail distributions exist across Africa, a continent-wide, harmonized database of intermediate-host snails is still lacking. We therefore join you in urging WHO and the wider research community to develop such a resource, which would markedly strengthen the biological realism of predictive models. We also recognize the emerging challenge posed by hybrid schistosome lineages (e.g., *S. haematobium* × *S. bovis* in Senegal and Cameroon). As georeferenced data on snail habitats and parasite strains become available, we will incorporate them so that ecological and evolutionary processes are explicitly represented. In the interim, we are partnering with parasitologists and drawing on regional snail surveys and environmental proxies to refine risk estimates at focal scales.

## Spatial resolution and future improvements.

It was correctly noted that a 5-km grid can miss micro-foci of transmission. This constraint reflects the spatial resolution of the satellite-derived covariates currently available. Advances in artificial-intelligence-based super-resolution, geostatistical down-scaling, and very-high-resolution remote-sensing products should soon allow sub-kilometer risk surfaces, and we are actively prototyping these approaches. Beyond spatial granularity, we acknowledge the importance of demographic disaggregation. Our present dataset lacks uniformly coded Water, Sanitation, and Hygiene indicators and age- or sex-stratified prevalence, but we are compiling such information for the next model iteration. Including community water-access data, sanitation coverage, and age/sex-specific infection rates will help identify hyper-local hotspots more accurately.

## Predictive equity and data gaps.

Training on data from only 12 countries risks under-representing the epidemiological diversity of sub-Saharan Africa. To close this gap, we are conducting a systematic literature review and collaborating with national control programs to assemble additional geo-referenced surveys from under-represented settings. In parallel, we are redesigning our modeling pipeline to be modular so that localized inputs (such as region-specific diagnostics, snail distribution layers, or behavioral-risk indices) can be integrated without rebuilding the entire architecture. We are also testing hybrid frameworks that couple Bayesian geostatistics with explainable machine learning (ML) ensembles, thereby combining ecological context with predictive power. Together, these data-expansion and architectural improvements aim to maximize the model’s transferability and fairness across diverse schistosomiasis-endemic regions.

We are grateful for the thoughtful engagement with our work and shared expertise in this field. This letter has stimulated important discussions about balancing technical innovation with practical implementation in global health settings.

Thank you again for the constructive feedback, which will undoubtedly help advance the application of ML in neglected tropical disease control.
